# Supplementary material for: Focal amplifications are associated with chromothripsis events and diverse prognoses in gastric cardia adenocarcinoma
Source: Nat Commun. 2021 Nov 11;12:6489. doi: 10.1038/s41467-021-26745-3 (PMC8586158; doi:10.1038/s41467-021-26745-3)
Supplement: Supplementary file 1 — Supplementary information [file 41467_2021_26745_MOESM1_ESM.pdf]

Supplementary information:

Supplementary Data 1: Sequencing quality assessment of whole genomic sequencing (WGS) data from 36 pairs of GCA tumour and adjacent normal tissues.

Supplementary Data 2: Detailed characterization of individual focal amplifications from the prediction of WGS data using AmpliconArchitect (AA).

Supplementary Data 3: Characterization of oncogene co-amplification in focal amplifications.

Supplementary Data 4: Clinicopathological records, microsatellite instability (MSI) staining, and chromosome instability (CIN) grade in 36 GCA tumours.

Supplementary Data 5: Sequencing quality assessment of whole exome sequencing (WES)data from 75 pairs of GCA tumour and adjacent normal tissues.

Supplementary Data 6: Clinicopathological records in 75 GCA patients with whole exome sequencing (WES)data.

Supplementary Data 7: *ERBB2* RNA expression and ERBB2 protein expression in 44 GCA patients.

Supplementary Data 8: Clinicopathological records and ERBB2 protein immunohistochemistry (IHC) staining in 1668 GCA patients.

Source Data: original data for each panel of figures.

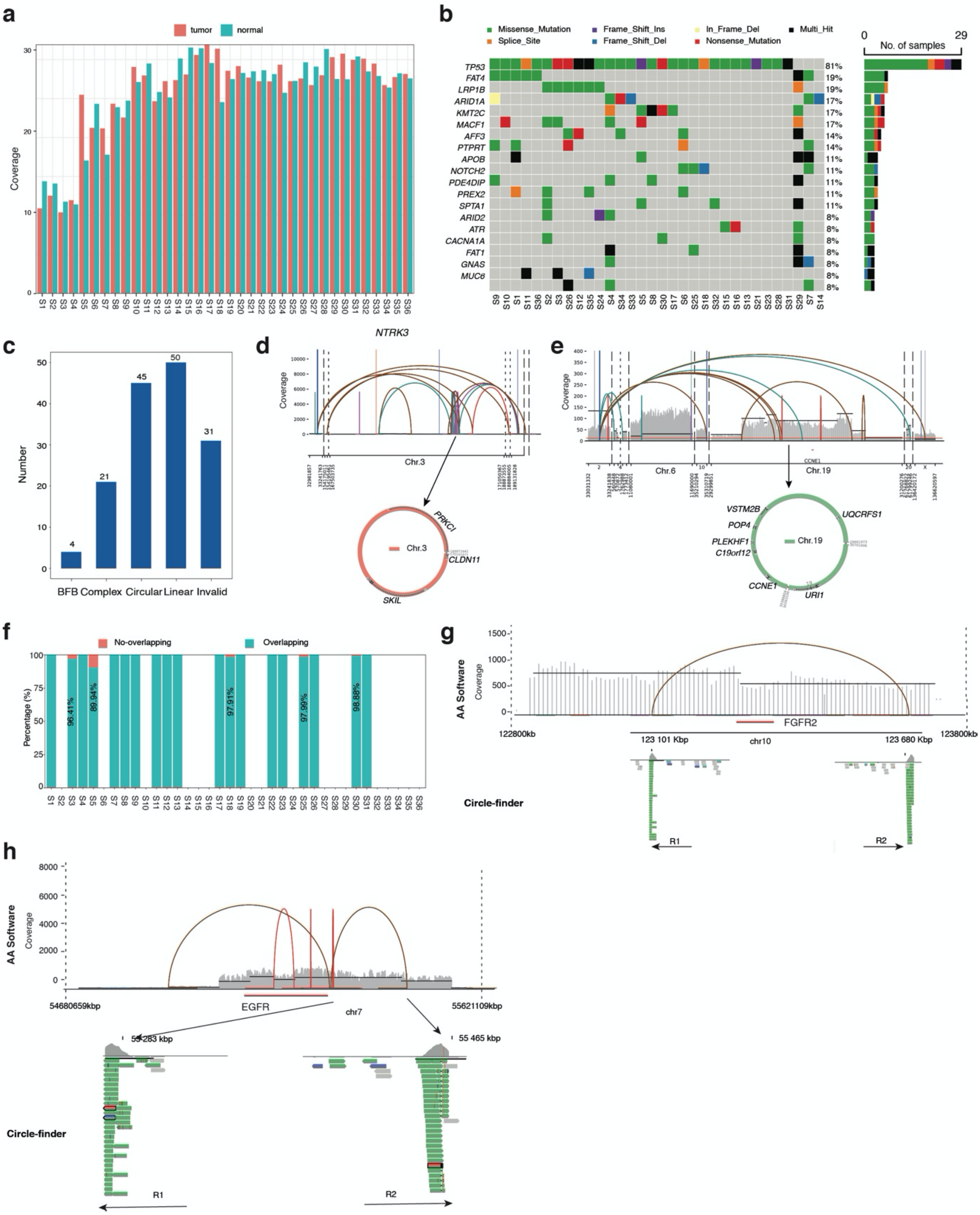

Supplementary Figure 1: Prediction of focal amplifications from the Chinese GCA cohort using whole genomic sequencing (WGS).

a, Sequencing depth of WGS from 36 (S1-S36) pairs of GCA tumour and adjacent normal tissue. Tumour = GCA tumour tissue; Normal = adjacent normal tissue.

b, Mutation frequency of cancer driver-genes from 36 (S1-S36) GCA patients.

c, Number of different types of focal amplifications predicted by AmpliconArchitect (AA) software in the 36 GCA cohort, where focal amplifications were further classified into circular

(ecDNA), complex, linear, breakage-fusion bridge (BFB) and invalid.

d and e, Examples of circular focal amplifications predicted by AmpliconArchitect (AA) software and constructed into circular format.

f, Summary of overlapping frequency in circular focal amplifications from prediction of AmpliconArchitect (AA) and detection using Circle-finder. The empty bar represents no ecDNA or no circular focal amplifications identified by AA.

g, h, Example of circular focal amplifications detected by both AA software and Circle-finder. Bottom panel: The location of forward and reverse sequencing reads (R1 = read 1, R2 = read 2) in the circular junction point are indicated on the genome browser.

Source data are provided as a Source Data file for Supplementary Figure 1a-c.

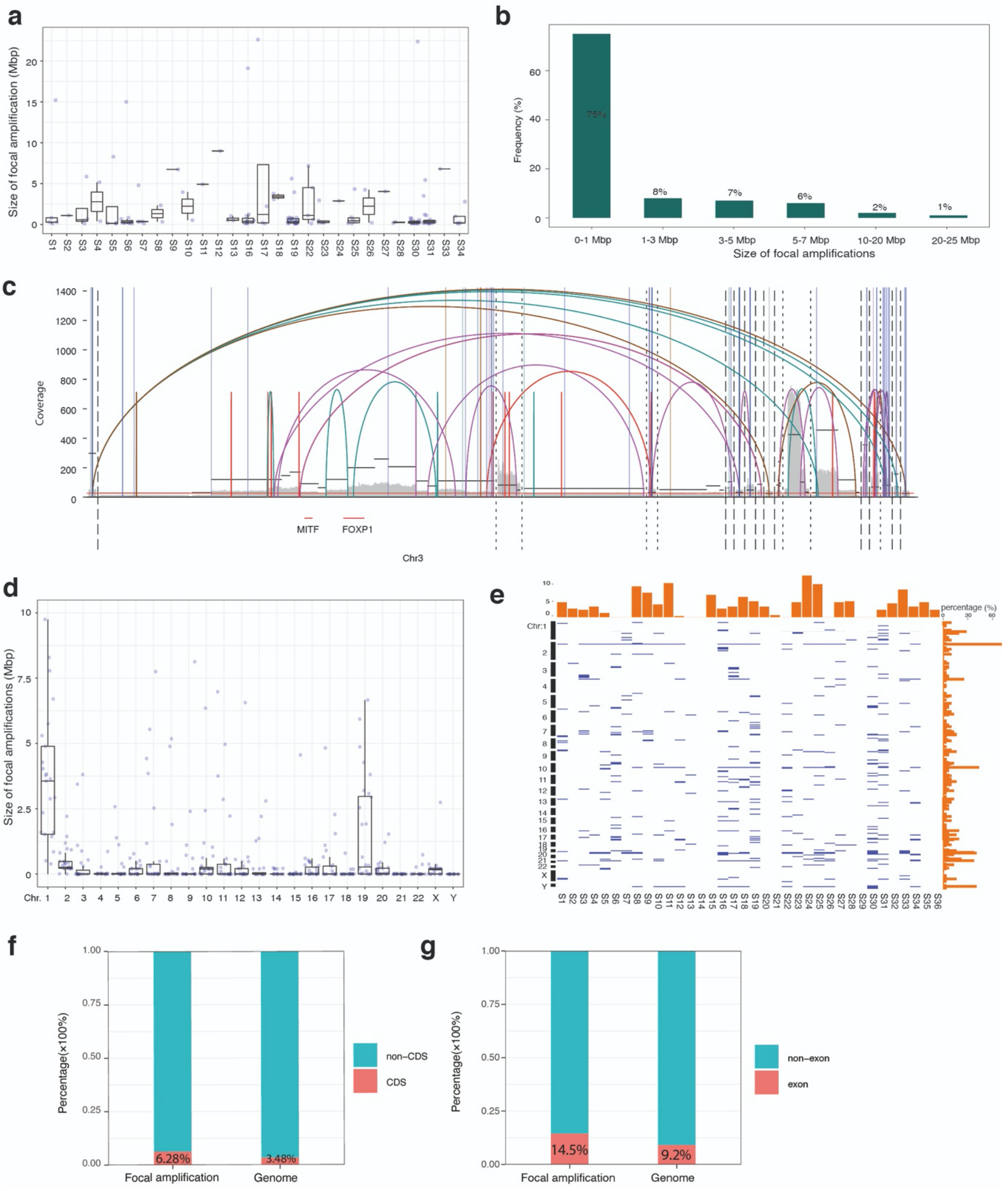

Supplementary Figure 2: Characterization of focal amplifications in the GCA cohort.

a, Summary of focal amplifications size in each GCA patient, where each dot represents one focal amplification (The number of focal amplifications, *n*=5, *n*=1, *n*=4, *n*=2, *n*=4, *n*=12, *n*=5, *n*=2, *n*=1, *n*=2, *n*=1, *n*=1, *n*=3, *n*=0, *n*=0, *n*=11, *n*=4, n=2, *n*=24, *n*=0, *n*=0, *n*=5, *n*=6, *n*=1, *n*=6, *n*=2, *n*=1, *n*=2, *n*=0, *n*=19, *n*=19, *n*=0, *n*=1, *n*=5, *n*=0, *n*=0 for S1 to S34). The box plots show the minima (bottom dot), the maxima (top dot), the median (middle line) and the first

and third quartiles (boxes), whereas the whiskers show 1.5x the interquartile range IQR above and below the box.

b, Size distribution of focal amplifications in our GCA cohort.

c, Example of large focal amplifications (> 20 Mbp) deconvoluted into multiple potential combinations of amplicons using AA software, where different connection lines on the top represent potential combinations within focal amplifications regions.

d, Summary of focal amplifications size in each chromosome from the cohort. Each dot represents one focal amplification (The number of biologically independent samples, n = 28). The box plots show the minima (bottom dot), the maxima (top dot), the median (middle line) and the first and third quartiles (boxes), whereas the whiskers show 1.5× the interquartile range IQR above and below the box.

e, Distribution of focal amplifications in each chromosome and each patient in the cohort.

f, Occupancy comparison of coding sequence (CDS) and noncoding sequence (non-CDS) regions of focal amplifications and the whole genome, where the percentage was calculated as follows: length of CDS or non-CDS regions divided by the total length of focal amplifications regions or whole genome.

g, Occupancy comparison of exon and non-exon regions of focal amplifications and the whole genome, where the calculation strategy is the same as in f.

Source data are provided as a Source Data file for Supplementary Figure 2a, b, d-g.

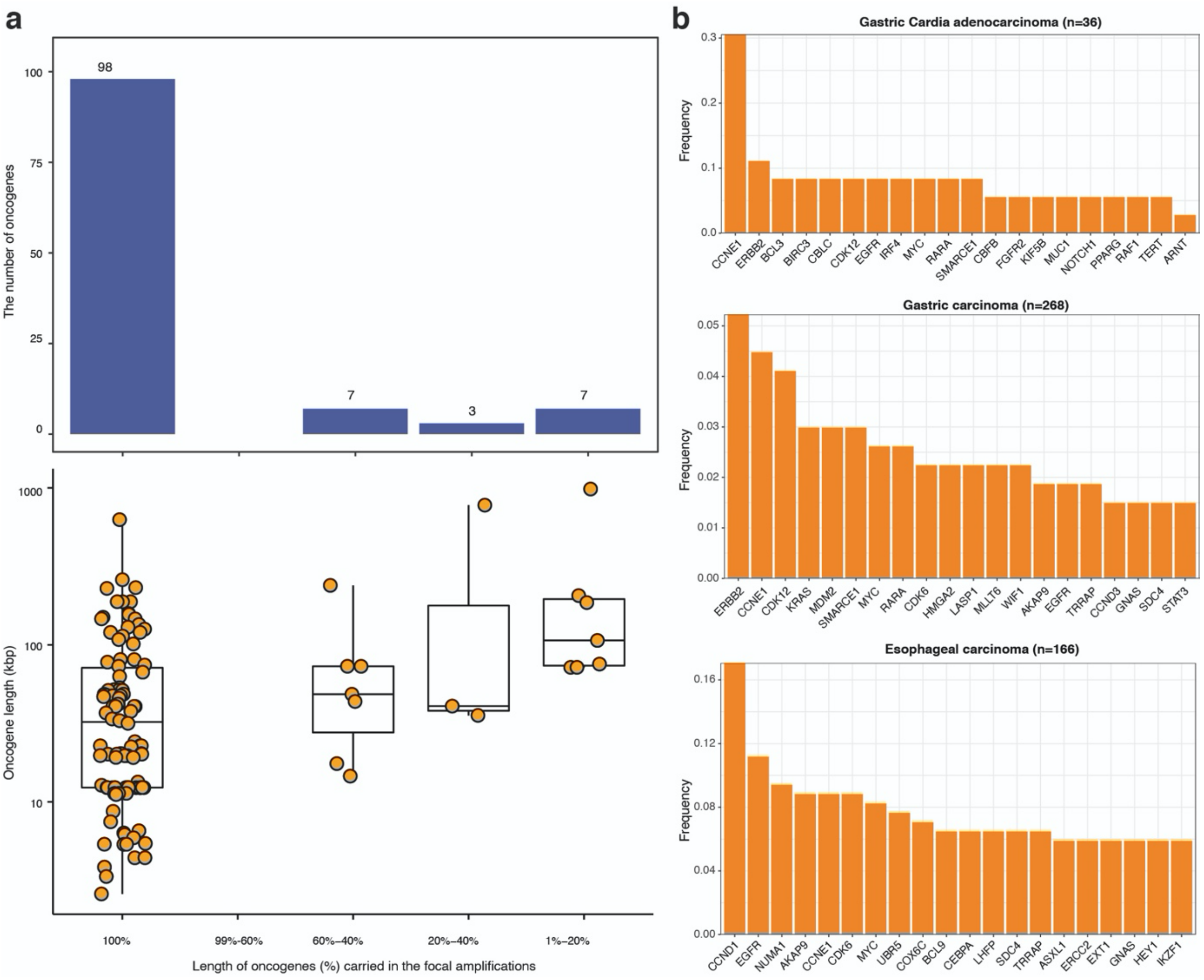

Supplementary Figure 3:

a, Characterisation of oncogenes carried in focal amplifications. Top: number of oncogenes; bottom: length of oncogenes (%) carried in the focal amplifications (*n*=98, *n*=0, *n*=7, *n*=3, *n*=7 for the group of 100%, 99%-60%, 60%-40%, 40%-20% and 1%-20%). The box plots show the minima (bottom dot), the maxima (top dot), the median (middle line) and the first and third quartiles (boxes), whereas the whiskers show 1.5× the interquartile range IQR above and below the box.

b, Comparison of lists of oncogene focal amplifications detected in our GCA cohort, TCGA gastric carcinoma and TCGA esophageal carcinoma.

Source data are provided as a Source Data file for Supplementary Figure 3a-c.

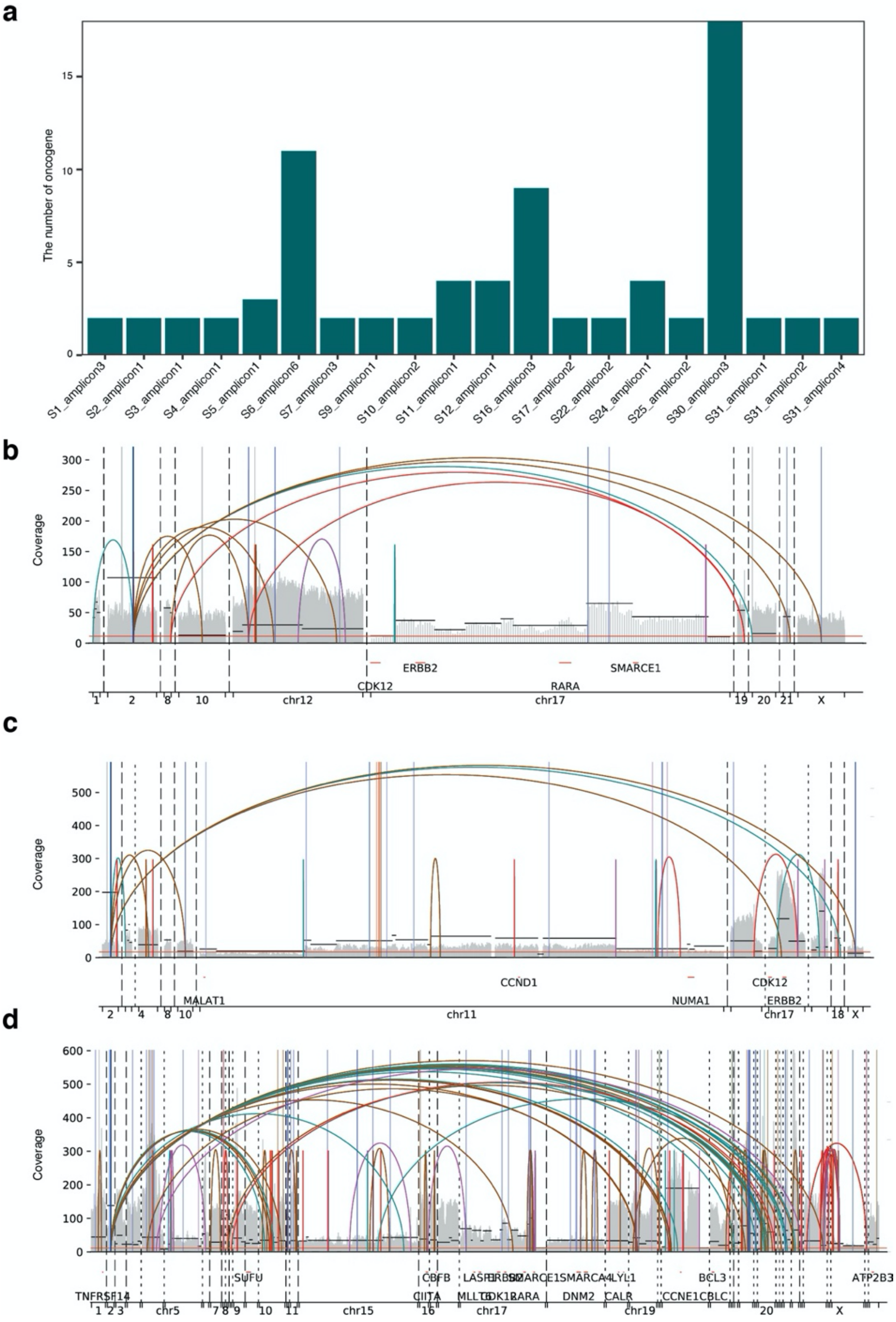

Supplementary Figure 4: Oncogene focal amplifications coamplification in GCA.

a, Number of oncogenes in each focal amplifications. S = Sample. Examples of oncogene focal amplifications co-amplification in our cohort: **b,** (including the following genes: *CDK12*, *ERBB2*, *RARA*, and *SMARCE1*), **c,** (including the following genes: *CCND1*, *NUMA1*, *CDK12* and *ERBB2*), and **d,** (including the following genes: *ERBB2*, *RARA*, *CCNE1*, *LYL1*, *CDK12*, *CIITA*, *MLLT6*, *DNM2*, *TNFRSF14*, *CBFB*, *SMARCE1*, *SUFU*, LASP1, *CBLC*, *ATP2B3*, *SMARCA4*, *CALR*, *BCL3*). Source data are provided as a Source Data file for Supplementary Figure 4a.

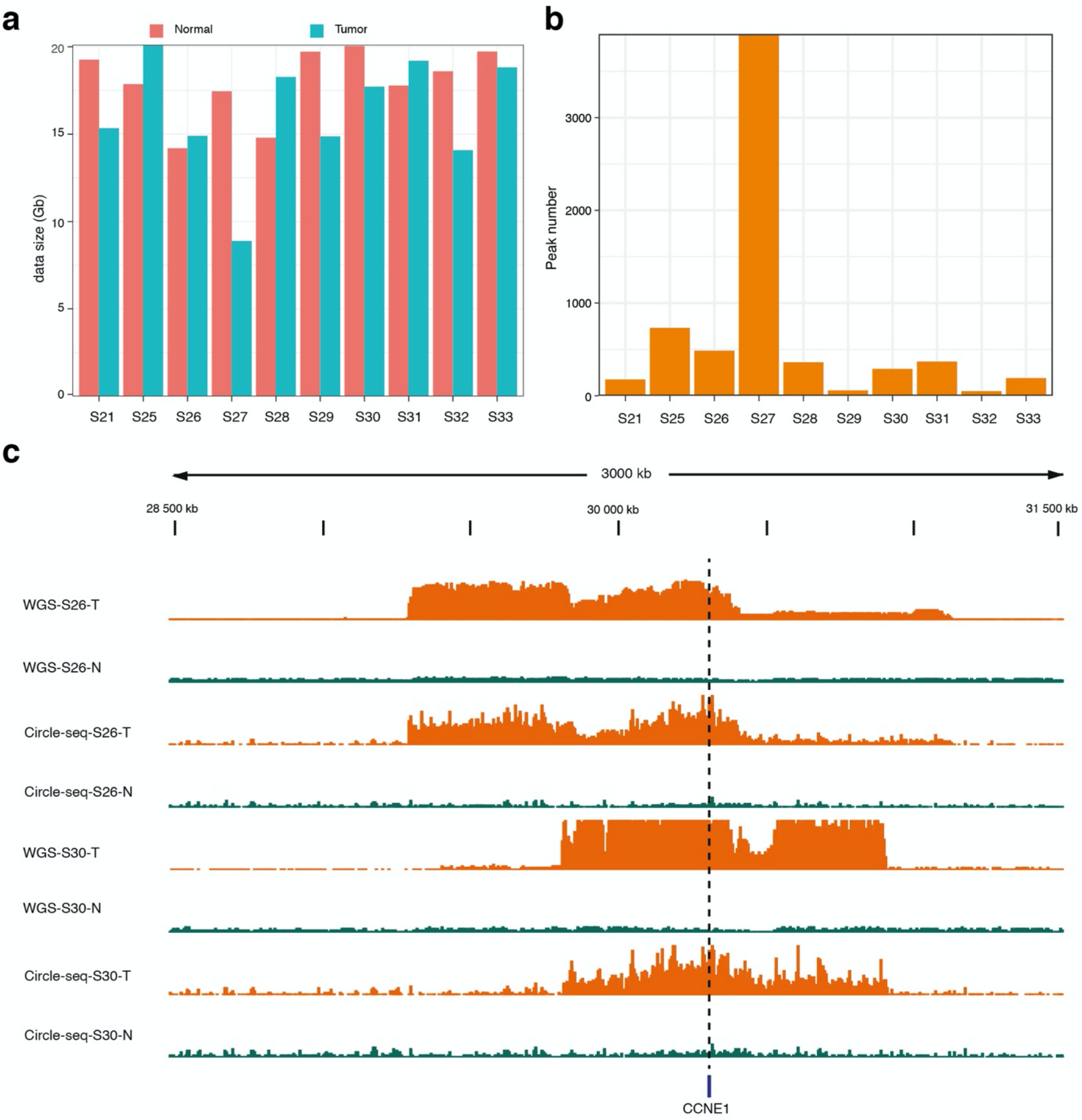

Supplementary Figure 5: Validation of circular focal amplifications using Circle-seq.

a, Sequencing coverage summary of Circle-seq from GCA patients (n = 10). Tumour = GCA tumour tissue; Normal = adjacent normal tissue.

b, Summary of peak numbers from Circle-seq for each GCA patient. Each peak represents one DNA fragment in the circular DNA.

c, Genome browser track at the *CCNE1* gene from whole genome sequencing (WGS) and Circle-seq. The dotted line indicates the location of the *CCNE1* gene. N = adjacent normal tissue, T = GCA tumour tissue.

Source data are provided as a Source Data file for Supplementary Figure 5a-b.

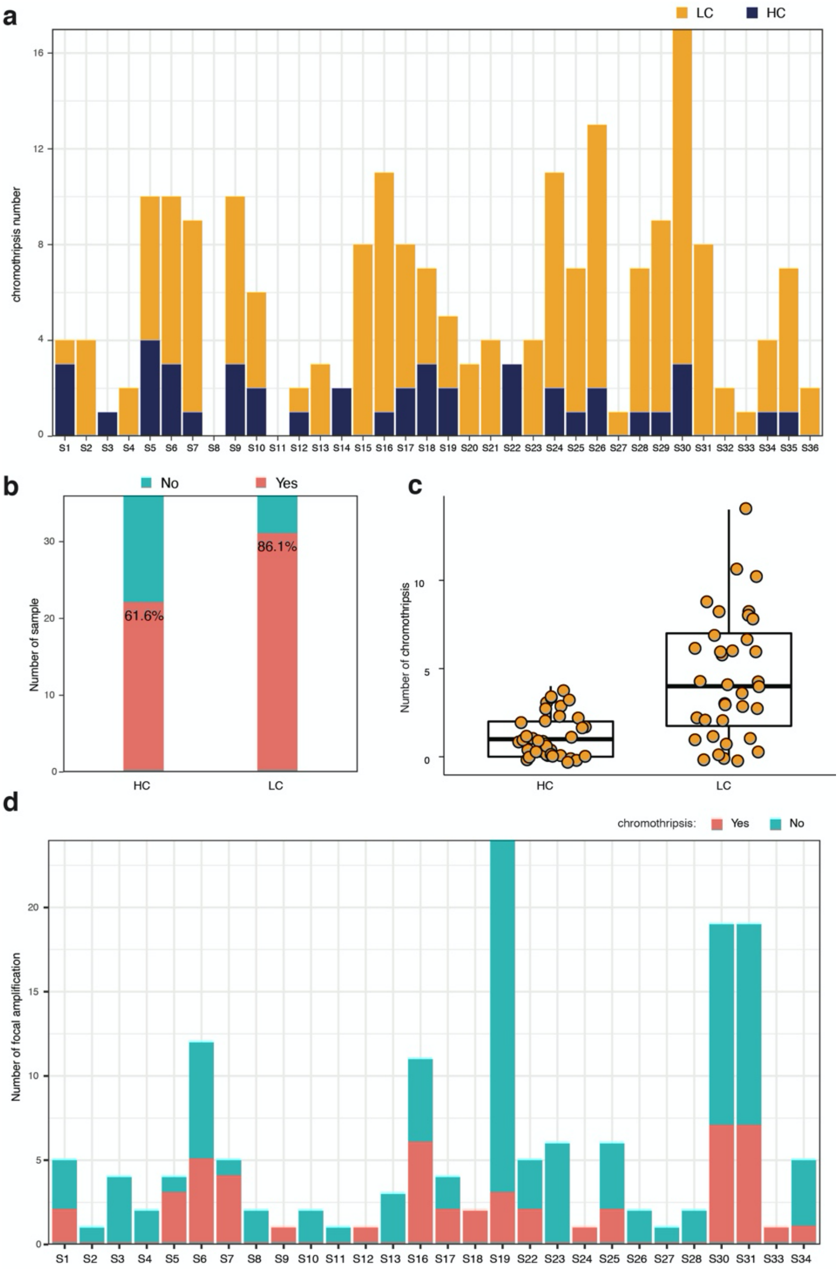

Supplementary Figure 6: **Detection of chromothripsis in the GCA cohort.**

a, Summary of predicted chromothripsis events in the GCA cohort using different parameters.HC = high confidence chromothripsis, LC = low confidence chromothripsis.

b, Frequency of chromothripsis detected in the GCA cohort. HC = high confidence chromothripsis, LC = low confidence chromothripsis.

c, Range of chromothripsis events detected from the high confidence (HC) and low confidence(LC) samples in the GCA cohort (The number of biologically independent samples, *n*=22 for HC, *n*=31 for LC). The box plots show the minima (bottom dot), the maxima (top dot), the median (middle line) and the first and third quartiles (boxes), whereas the whiskers show 1.5× the interquartile range IQR above and below the box.

d, Summary of chromothripsis events occurring at regions of focal amplifications in our GCA cohort.

Source data are provided as a Source Data file for Supplementary Figure 6a-d.

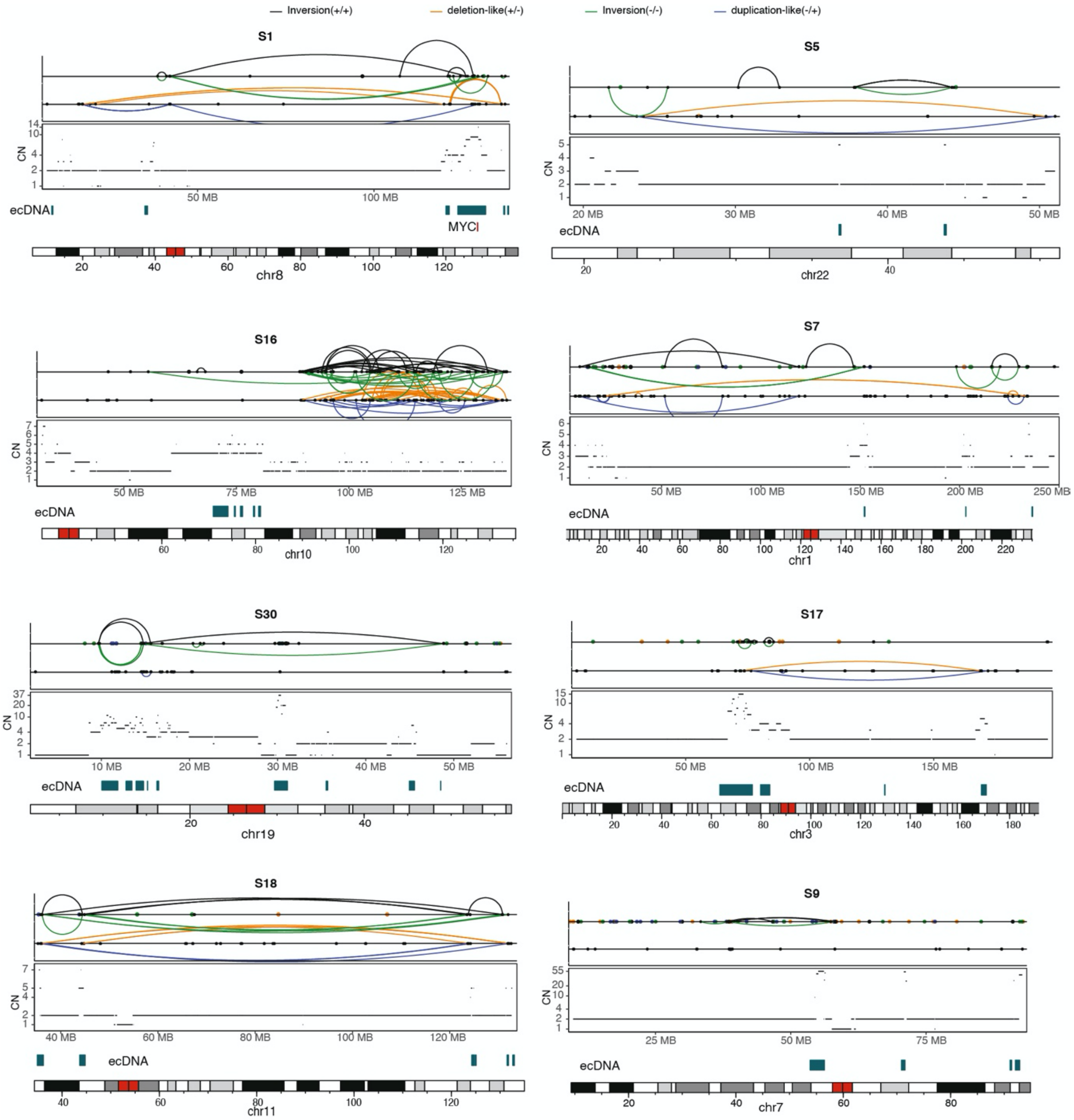

Supplementary Figure 7: **The fine structure of detected chromothripsis at different focal amplifications regions in different GCA patients.** S = Sample**.**

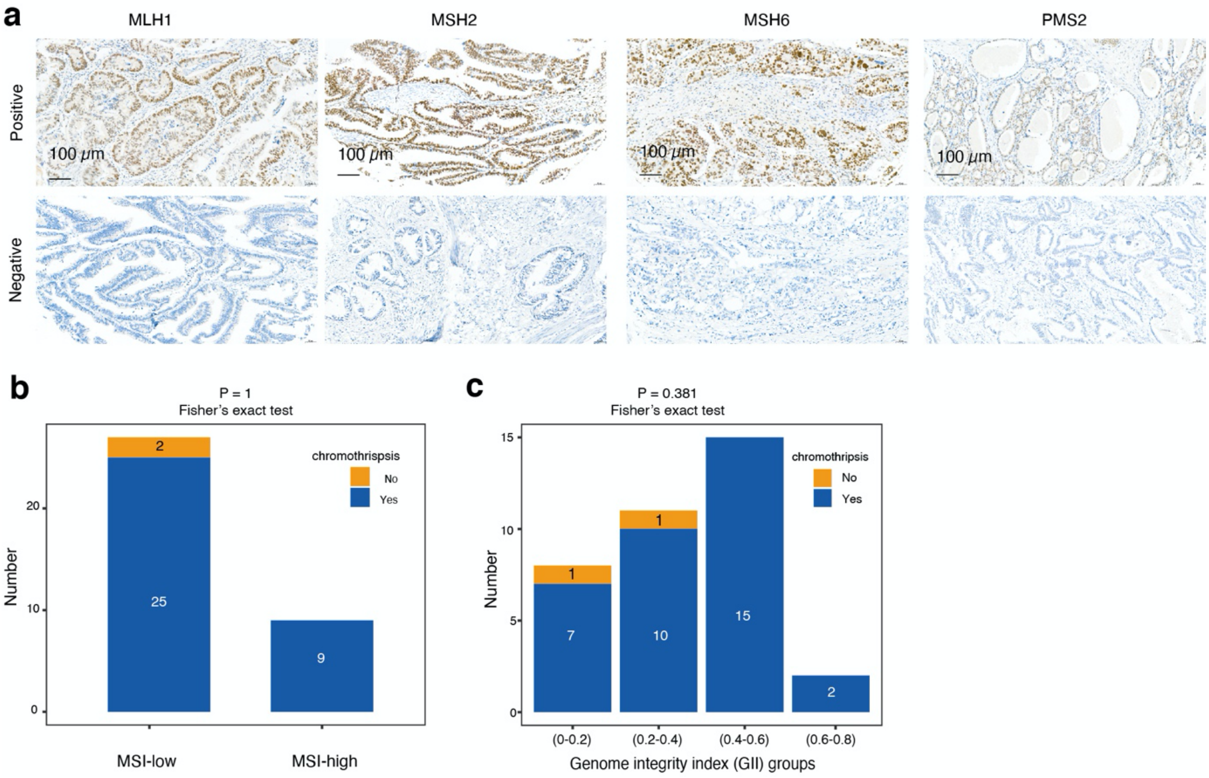

Supplementary Figure 8: Microsatellite instability (MSI), chromosome instability (CIN) and chromothripsis events.

a, Representative immunohistochemistry (IHC) images of four proteins (MLH1, MSH2, MSH6 and PMS2) from 36 patients. If one of four proteins exhibited negative staining by IHC, we labelled the patient as MSI-high. If all four proteins exhibited positive staining by IHC, we labelled the patient as MSI-low.

b, Presence and absence of chromothripsis events in the MSI-high and MSI-low groups of GCA patients. The numbers on the bars are patient numbers. The p-value was calculated using the two-sided Fisher exact test.

Source data are provided as a Source Data file for Supplementary Figure 8b-c.

c, Comparison of chromothripsis events in different groups of chromosome instability (CIN), where CIN was divided into 4 groups based on different genome integrity indices (GIIs). The numbers on the bars are patient numbers. The *p*-value was calculated using the two-sided Fisher exact test.

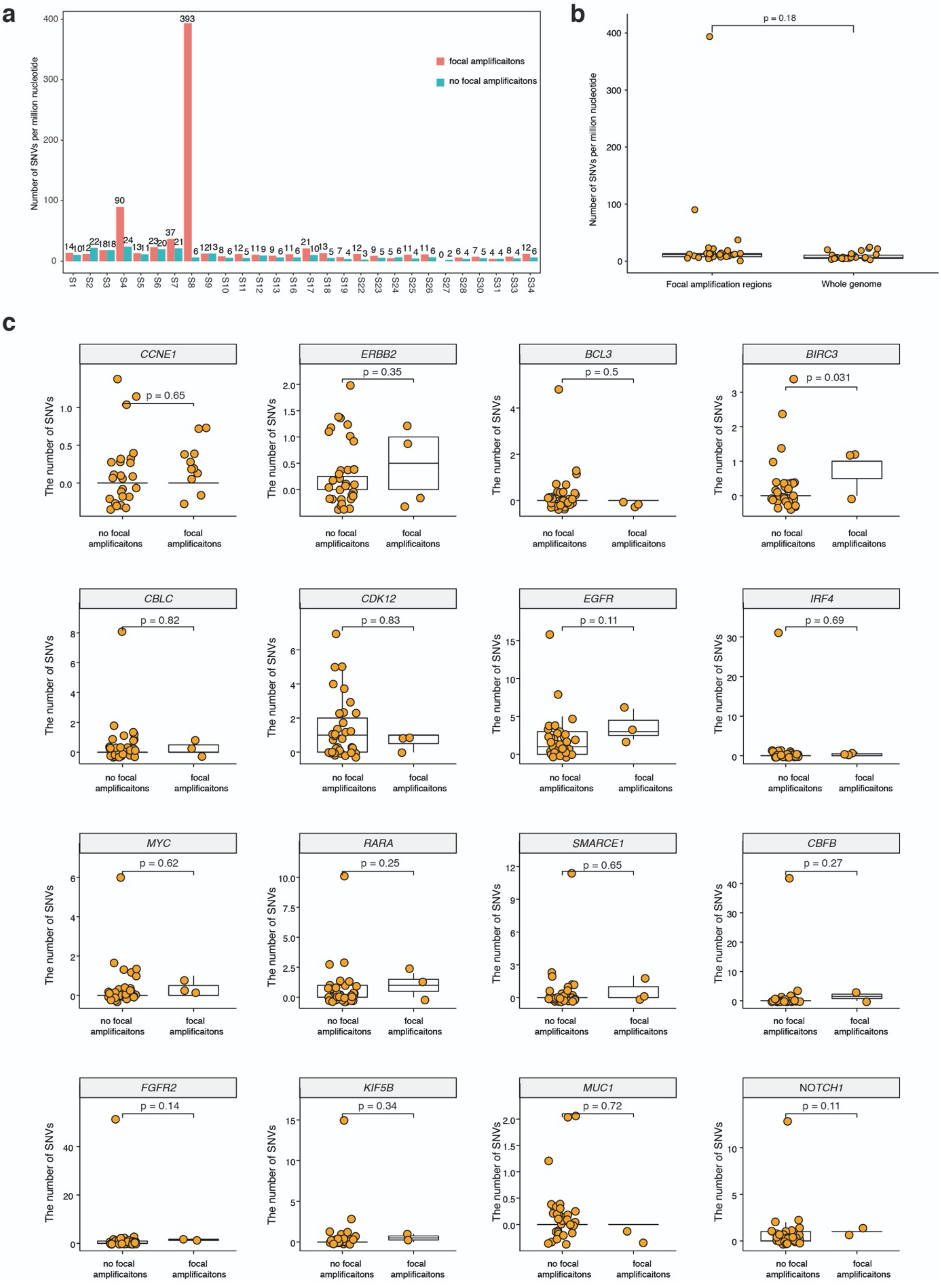

Supplementary Figure 9: **Somatic gene mutation and focal amplifications.**

a, Comparison of the average number of single-nucleotide variants (SNVs) per million nucleotides from regions of focal amplifications and from the whole genome in individual

patients. S = Sample.

b, Comparison of the average number of single-nucleotide variants (SNVs) per million nucleotides from regions of focal amplifications and from the whole genome in all 36 patients. Each dot represents one patient. The p-value was calculated using the two-sided Wilcoxon signed-rank test. (The number of biologically independent samples, *n*=36, *n*=36). The box plots show the minima (bottom dot), the maxima (top dot), the median (middle line) and the first and third quartiles (boxes), whereas the whiskers show 1.5× the interquartile range IQR above and below the box.

c, Comparison of SNV numbers on oncogenes between the focal amplifications-present and focal amplifications-absent groups in the cohort, where 16 oncogenes (*CDK12* are tumour suppressor gene but with oncogenic features) were selected, and focal amplifications were observed in at least two patients. Each dot represents one sample. The p-value was calculated using the two-sided Wilcoxon signed-rank test (The number of biologically independent samples, *n*=25, *n*=11 for CCNE1; *n*=32, *n*= 4 for *ERBB2*; *n*=33, *n*=3 for *BCL3*; *n*=33, *n*=3 for *BIRC3*; *n*=33, *n*=3 for *CBLC*; *n*=33, *n*=3 for *CDK12*; *c*; n=33, *n*=3 for *IRF4*; *n*=33, *n*=3 for *MYC*; *n*=33, *n*=3 for RARA; *n*=33, *n*=3 for *SMARCE1*; *n*=34, *n*=2 for *CBFB*; n=34, *n*=2 for *FGFR2*; *n*=34, *n*=2 for *KIF5B*; *n*=34, *n*=2 for *MUC1*; *n*=34, *n*=2 for *NOTCH1*). The box plots show the minima (bottom dot), the maxima (top dot), the median (middle line) and the first and third quartiles (boxes), whereas the whiskers show 1.5× the interquartile range IQR above and below the box.
Source data are provided as a Source Data file for Supplementary Figure 9a-c.

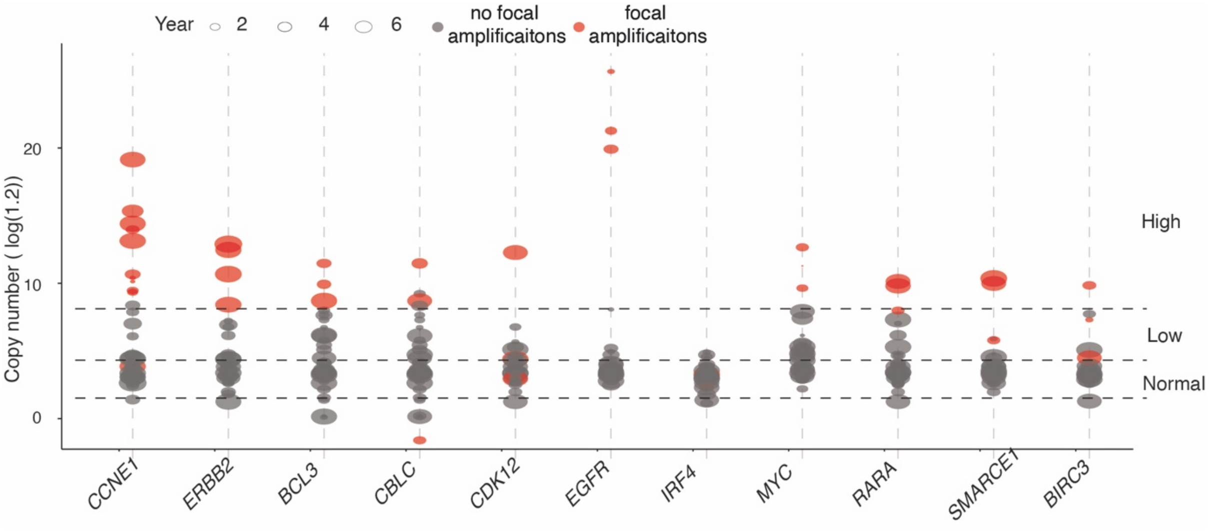

Supplementary Fig. 10: **Relationship between oncogenes, the presence of oncogene focal amplifications and patient prognosis in 36 GCA patients.** The copy numbers of oncogenes were divided into three groups: High, Low and Normal. High = high copy number of gene amplification, Low = low copy number of gene amplification, Normal = no gene amplification. *CDK12* is tumour suppressor gene but with oncogenic feature. Source data are provided as a Source Data file for Supplementary Figure 10.

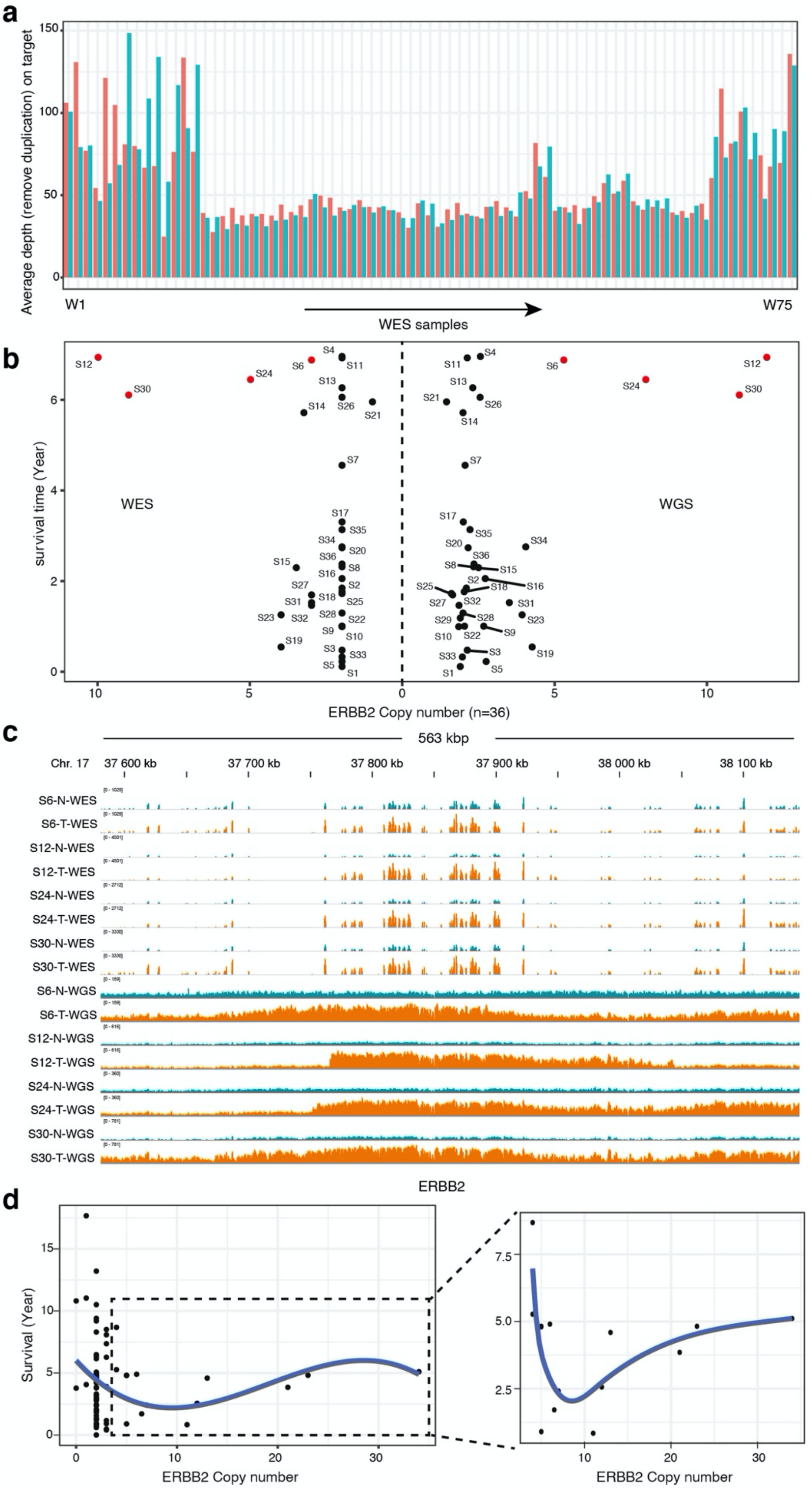

Supplementary Figure 11: **Correlation study of gene copy number from whole exome sequencing (WES) and patient prognosis**.

a, Sequencing coverage of WES from 75 pairs of GCA tumour and matched adjacent normal tissue. Tumour = GCA tumour tissue; Normal = adjacent normal tissue. S = Sample.
b, Comparison of the gene copy number of the *ERBB2* gene between WES and whole genome sequencing (WGS) in the same GCA samples. S = Sample.
c, Genome browser track of the *ERBB2* gene locus of both WES and WGS data from 4 pairs of GCA patients; -T- = GCA tumour tissue; N- = adjacent normal tissue.
d, Correlation of *ERBB2* gene copy number from WES and patient survival time in 75 GCA patients. Each dot represents one patient.
Source data are provided as a Source Data file for Supplementary Figure 11a, b, d.

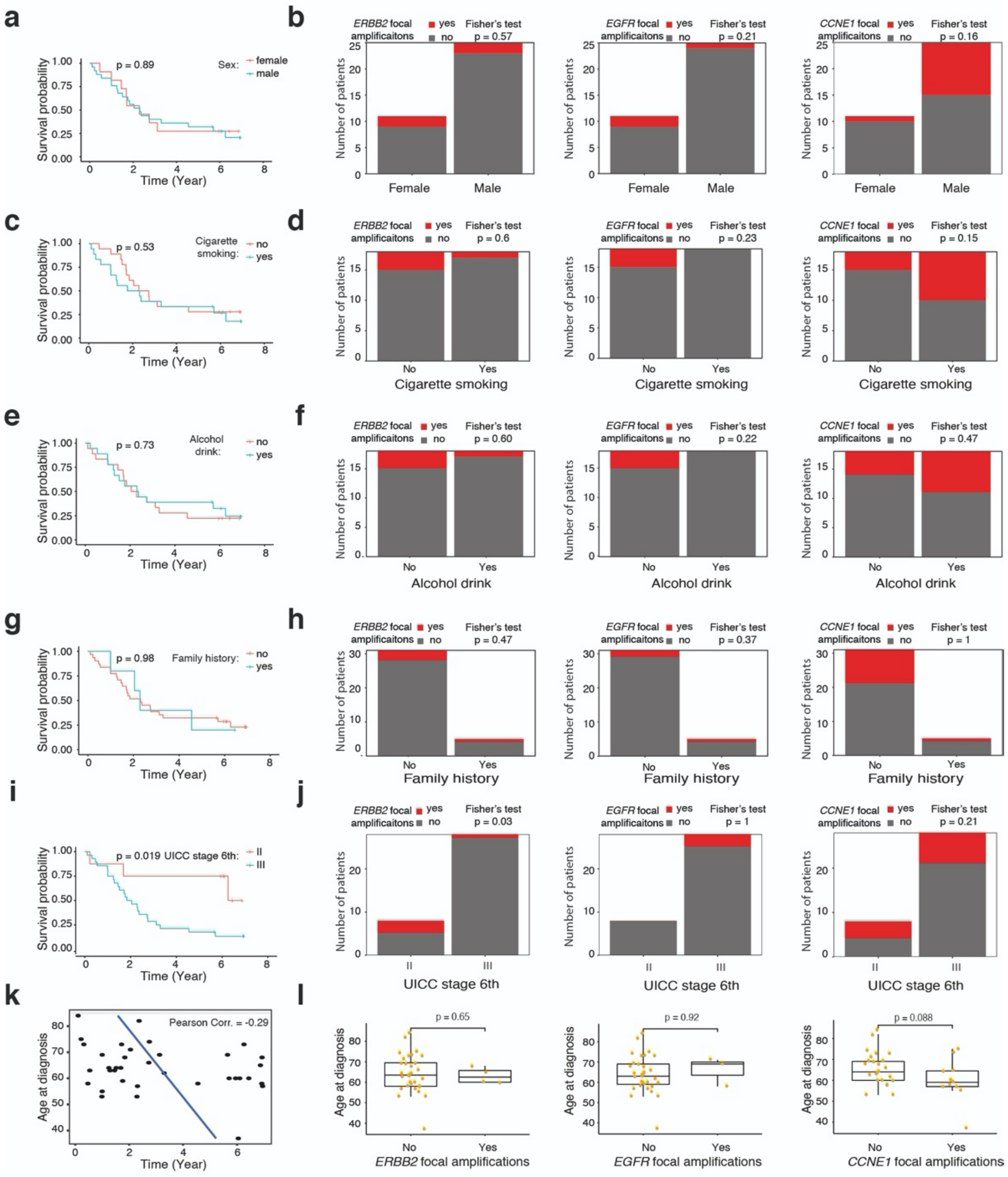

Supplementary Figure 12: **Prognostic analysis with clinicopathological variables and the presence of oncogene focal amplifications.**

a, Survival analysis of 36 GCA patients based on sex; p-value was calculated using the two-sided Log rank test.

b, Comparison of oncogene focal amplifications (ERBB2, EGFR, CCNE1) presence in different sex groups of 36 GCA patients; p-value was calculated using Fisher's test.

c, Survival analysis of 36 GCA patients based on smoking; p-value was calculated using the two-sided Log rank test.

d, Comparison of oncogene focal amplifications (ERBB2, EGFR, CCNE1) presence in smoking or non-smoking group of 36 GCA patients; p-value was calculated using the two-sided Fisher's exact test.

e, Survival analysis of 36 GCA patients based on alcohol consumption; p-value was calculated using the two-sided Log rank test.
f, Comparison of oncogene focal amplifications (ERBB2, EGFR, CCNE1) presence in drinking or non-drinking group of 36 GCA patients; p-value was calculated using the two-sided Fisher's exact test.
g, Survival analysis of 36 GCA patients based on family history; p-value was calculated using the two-sided log rank test.
h, Comparison of oncogene focal amplifications (ERBB2, EGFR, CCNE1) presence with family history or without family history in 36 GCA patients; p-value was calculated using the two-sided Fisher's test.
i, Survival analysis of 36 GCA patients based on the Union for International Cancer Control (UICC) tumour stage. The p-value was calculated using the two-sided log-rank test.
j, Comparison of oncogene focal amplifications (ERBB2, EGFR, CCNE1) presence in UICC tumour stages II and III of 36 GCA patients. The p-value was calculated using the two-sided Fisher's test.
k, The correlation of survival time and age at diagnosis in 36 GCA patients. Each dot represents one patient.
l, The comparison of age at diagnosis of GCA in absent and present oncogene focal amplifications (*ERBB2*, *EGFR*, *CCNE1*) groups of 36 GCA patients. The p-value was calculated using the two-sided Wilcoxon signed-rank test (The number of biologically independent samples, *n*=25, *n*=11 for *CCNE1*; *n*=25, *n*=11 for *CCNE1*; *n*=32, *n*= 4 for *ERBB2*; *n*=33; *n*=32, *n*= 4 for *ERBB2*;). The box plots show the minima (bottom dot), the maxima (top dot), the median (middle line) and the first and third quartiles (boxes), whereas the whiskers show 1.5× the interquartile range IQR above and below the box.
Source data are provided as a Source Data file for Supplementary Figure 12a-l.

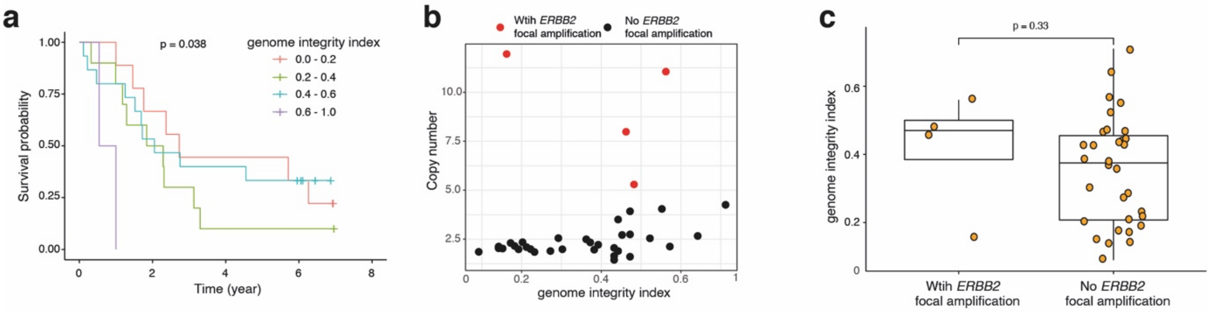

Supplementary Figure 13: **Prognostic analysis based on chromosomal instability (CIN).**

a, Survival analysis of 4 groups of CIN defined by the number of genome integrity indices (GIIs). The *p*-value was calculated using the two-sided log-rank test.

b, Scatter plot showing the relationship of ERBB2 gene copy number, presence of *ERBB2* focal amplifications (colour-coded) and CIN grade of patients, where each dot represents one patient.

c, Comparison of genome integrity index values between *ERBB2* focal amplifications present and absent groups. Each dot represents one sample. The p-value was calculated using the two-sided Wilcoxon signed-rank test (The number of biologically independent samples, *n*=4, *n*=32). The box plots show the minima (bottom dot), the maxima (top dot), the median (middle line) and the first and third quartiles (boxes), whereas the whiskers show 1.5× the interquartile range IQR above and below the box.

Source data are provided as a Source Data file for Supplementary Figure 13a-c.

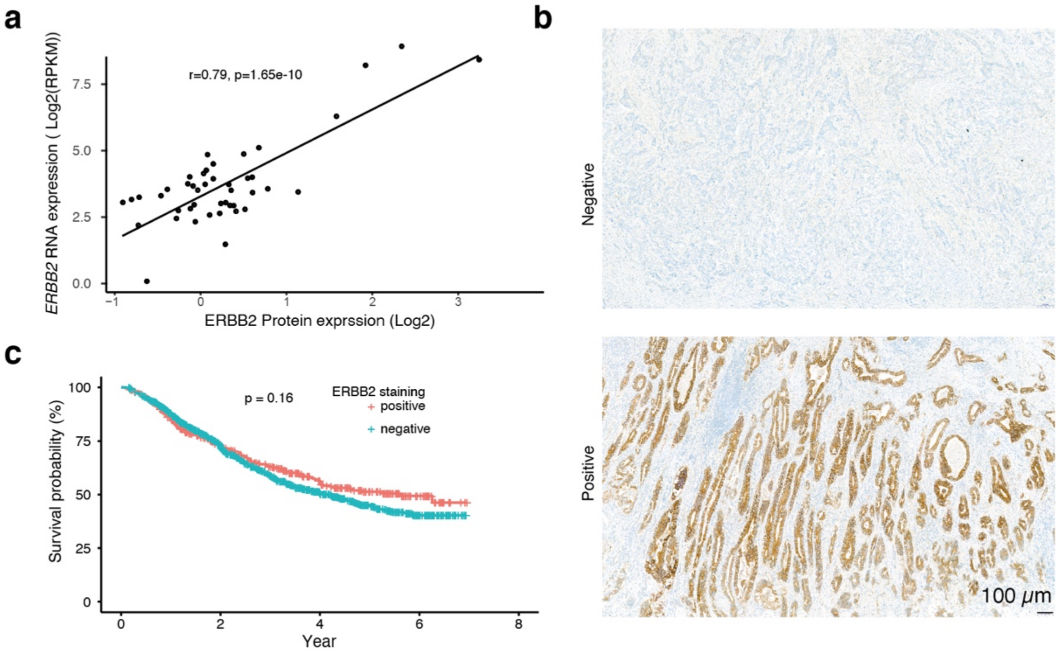

Supplementary Figure 14: ERBB2 protein staining and patient prognosis.

a, Scatter plot showing that there is a positive correlation between *ERBB2* RNA expression level and ERBB2 protein expression level in 44 GCA patients. Each dot represents one patient. The correlation and *p*-value were calculated using the two-sided Pearson correlation test.

b, Representative ERBB2 protein immunohistochemistry (IHC) images of positive (bottom) and negative (top) staining from 1668 GCA patients. For each IHC staining, the experiments were repeated three times as technical replicates.

c, Survival analysis of positive and negative *ERBB2* IHC staining groups in 1668 GCA patients. The *p*-value was calculated using the two-sided Rényi test (with Fleming Harrington (p = 1, q = 1).
