# Supplementary material for: Focal amplifications are associated with chromothripsis events and diverse prognoses in gastric cardia adenocarcinoma
Source: Nat Commun. 2021 Nov 11;12:6489. doi: 10.1038/s41467-021-26745-3 (PMC8586158; doi:10.1038/s41467-021-26745-3)
Supplement: Supplementary file 11 — Reporting Summary [file 41467_2021_26745_MOESM11_ESM.pdf]

nature research

Corresponding author(s): Xingqi Chen, Li-dong Wang

Last updated by author(s): Oct 18, 2021

Reporting Summary

Nature Research wishes to improve the reproducibility of the work that we publish. This form provides structure for consistency and transparency in reporting. For further information on Nature Research policies, see our Editorial Policies and the Editorial Policy Checklist.

Statistics

For all statistical analyses, confirm that the following items are present in the figure legend, table legend, main text, or Methods section.

n/a	Confirmed	
☐	☒	The exact sample size (*n*) for each experimental group/condition, given as a discrete number and unit of measurement
☐	☒	A statement on whether measurements were taken from distinct samples or whether the same sample was measured repeatedly
☐	☒	The statistical test(s) used AND whether they are one- or two-sided<br>*Only common tests should be described solely by name; describe more complex techniques in the Methods section.*
☒	☐	A description of all covariates tested
☐	☒	A description of any assumptions or corrections, such as tests of normality and adjustment for multiple comparisons
☐	☒	A full description of the statistical parameters including central tendency (e.g. means) or other basic estimates (e.g. regression coefficient) AND variation (e.g. standard deviation) or associated estimates of uncertainty (e.g. confidence intervals)
☐	☒	For null hypothesis testing, the test statistic (e.g. *F*, *t*, *r*) with confidence intervals, effect sizes, degrees of freedom and *P* value noted<br>*Give P values as exact values whenever suitable.*
☒	☐	For Bayesian analysis, information on the choice of priors and Markov chain Monte Carlo settings
☒	☐	For hierarchical and complex designs, identification of the appropriate level for tests and full reporting of outcomes
☐	☒	Estimates of effect sizes (e.g. Cohen's *d*, Pearson's *r*), indicating how they were calculated

Our web collection on statistics for biologists contains articles on many of the points above.

Software and code

Policy information about availability of computer code

Data collection	No code was used to collect data in this study - code was only used for data analysis.

Data analysis

AmpliconArchitect software (https://github.com/virajbdeshpande/AmpliconArchitect);
AmpliconClassifier codes (https://github.com/jluebeck/AmpliconClassifier);
bwa v0.7.17 software (http://bio-bwa.sourceforge.net/);
samtools 1.9 software (http://www.htslib.org/);
bedtools v2.29.2 software (https://github.com/arq5x/bedtools2);
ggplot2 v3.3.5 package (https://cran.r-project.org/web/packages/ggplot2/index.html);
Shatterseek package (https://github.com/parklab/ShatterSeek);
Copycat package (https://github.com/chrisamiller/copyCat);
IGV v2.9.4 software (https://software.broadinstitute.org/software/igv/);
maftools v3.13 package (https://bioconductor.org/packages/release/bioc/html/maftools.html);
novoBreak_distribution_v1.1.3 software (https://github.com/abjonnes/novoBreak);
bcftools v1.9 software (https://samtools.github.io/bcftools/bcftools.html);
delly v0.8.1 software (https://github.com/dellytools/delly);
gatk v4.1.7 software (https://gatk.broadinstitute.org/hc/en-us);
circos v0.69-8 software (http://circos.ca/);
homer v4.11 software (http://homer.ucsd.edu/homer/motif/);
survmine v0.4.9 package (https://cran.r-project.org/web/packages/survminer/index.html);
survival v3.2-13 package (https://cran.r-project.org/web/packages/survival/index.html);
survMisc v0.5.5 package (https://cran.r-project.org/web/packages/survMisc/index.html);
Custom codes are available at https://github.com/pengweixing/ecDNA-on-GCA;

For manuscripts utilizing custom algorithms or software that are central to the research but not yet described in published literature, software must be made available to editors and reviewers. We strongly encourage code deposition in a community repository (e.g. GitHub). See the Nature Research guidelines for submitting code & software for further information.

Data

Policy information about availability of data

All manuscripts must include a data availability statement. This statement should provide the following information, where applicable:

- Accession codes, unique identifiers, or web links for publicly available datasets
- A list of figures that have associated raw data
- A description of any restrictions on data availability

The raw data of WGS data, WES data and Circle-Seq data generated in this study have been deposited in the China National Center for Bioinformation under accession code HRA000814 [https://ngdc.cncb.ac.cn/gsa-human/s/FqmW3jHX] .

Field-specific reporting

Please select the one below that is the best fit for your research. If you are not sure, read the appropriate sections before making your selection.

- [x] Life sciences
- [ ] Behavioural & social sciences
- [ ] Ecological, evolutionary & environmental sciences

For a reference copy of the document with all sections, see nature.com/documents/nr-reporting-summary-flat.pdf

Life sciences study design

All studies must disclose on these points even when the disclosure is negative.

Sample size

This study aims to explore the focal amplifications, ecDNAs, chromothripsis and SNVs in somatic GCA tumor tissue. Sample size was determined by the availability of tissue and cost of the experiment. Hence, no calculation to predetermine sample-size was performed. Therefore, we collected 36 pairs of GCA tumor samples and matched adjacent normal tissues for WGS, and 75 pairs of GCA tumor samples and matched adjacent normal tissues for WES. All of these sample are from from Esophageal Cancer database (1973-2020) which established and maintained by Henan Key Laboratory for Esophageal Cancer Research of the First Affiliated Hospital, Zhengzhou University.

Data exclusions

No data was excluded.

Replication

Our study investigated the genomic amplifications and mutations on human tumor tissues, the replication does not apply in this study. Sequencing replicates are not normally used for WGS data as each of them have enough sequencing coverage.

Randomization

The randomization does not apply in this study.

Blinding

This is an identification and association study,so samples were not blinded.

Reporting for specific materials, systems and methods

We require information from authors about some types of materials, experimental systems and methods used in many studies. Here, indicate whether each material, system or method listed is relevant to your study. If you are not sure if a list item applies to your research, read the appropriate section before selecting a response.

Materials & experimental systems

n/a	Involved in the study
☐	☒ Antibodies
☒	☐ Eukaryotic cell lines
☒	☐ Palaeontology and archaeology
☒	☐ Animals and other organisms
☐	☒ Human research participants
☒	☐ Clinical data
☒	☐ Dual use research of concern

Methods

n/a	Involved in the study
☒	☐ ChIP-seq
☒	☐ Flow cytometry
☒	☐ MRI-based neuroimaging

Antibodies

Antibodies used	anti-gammH2AX (1: 200 dilution, SAB5700329, Sigma-Aldrich); anti-MLH1 (1: 100 dilution, PA5-32497, Thermo Fisher Scientific); anti-MSH2 (1: 500 dilution, MA5-15740, Thermo Fisher Scientific); anti-MSH6 (1: 100 dilution, MA5-32040, Thermo Fisher Scientific); anti-PMS2 (1: 150 dilution, MA5-26269, Thermo Fisher Scientific); anti-ERBB2 (1:100 dilution, SAB5700151, Sigma-Aldrich)
Validation	Each antibody specificity was validated by the manufacture, and the validation result is posted on their website.

Human research participants

Policy information about studies involving human research participants

Population characteristics	All patients in our study were not received radiotherapy or chemotherapy before the surgery. 1668 GCA patients for ERBB2 immunohistochemistry (IHC) staining are from the Esophageal Cancer database (from years of 1973-2020) which established and maintained by Henan Key Laboratory for Esophageal Cancer Research of the First Affiliated Hospital, Zhengzhou University, China.
Recruitment	The patients in this study are recruited as volunteers by An Yang cancer hospital, China and the First Affiliated Hospital, Zhengzhou University, China under the guidance of ethic permission. All patients were informed in our study with a consent document signed, and the effect that consent to publish clinical information potentially identifying individuals was obtained. The recruitment procedure of patients is not from any biased-selection.
Ethics oversight	The ethical research committees at An Yang cancer hospital, China and the First Affiliated Hospital, Zhengzhou University, China approved the study.

Note that full information on the approval of the study protocol must also be provided in the manuscript.
